# Multifocal Early-Onset Neonatal Listeriosis with Discordant GradientStrip Ampicillin Non-Susceptibility: A Case Report

**DOI:** 10.3390/pathogens15070674

**Published:** 2026-06-26

**Authors:** Elena Teona Cosovanu, Silvia Ionescu, Eric Oliviu Cosovanu, Costin Damian, Bogdan Aurelian Stana, Ecaterina Iftime, Antoneta Dacia Petroaie, Tiberiu Lunguleac, Ileana Katerina Ioniuc, Elena Adorata Coman, Cristina Daniela Dimitriu, Demetra Gabriela Socolov, Luminita Smaranda Iancu, Irina Draga Caruntu, Ramona Gabriela Ursu

**Affiliations:** 1 “Grigore T. Popa” University of Medicine and Pharmacy, 16 Universitatii Street, 700115 Iaşi, Romaniaantoneta.petroaie@umfiasi.ro (A.D.P.); tiberiu.lunguleac1@umfiasi.ro (T.L.); ileana.ioniuc@umfiasi.ro (I.K.I.); luminita.iancu@umfiasi.ro (L.S.I.); irina.caruntu@umfiasi.ro (I.D.C.);; 2 Medical Analysis Laboratory, Clinical Hospital of Pneumology, 700115 Iaşi, Romania; 3 “Cuza-Vodă” Clinical Hospital of Obstetrics and Gynecology, 700038 Iaşi, Romania; 4 Iaşi Regional Center for Public Health, National Institute of Public Health, 700465 Iaşi, Romania

**Keywords:** *Listeria monocytogenes*, neonatal listeriosis, early-onset neonatal sepsis, premature infant, vertical transmission, ampicillin non-susceptibility, antimicrobial susceptibility testing, case report

## Abstract

Background: Early-onset neonatal listeriosis is a rare, life-threatening infection of vertical origin caused by *Listeria monocytogenes*. First-line therapy is intravenous ampicillin combined with an aminoglycoside; acquired β-lactam resistance is exceptionally uncommon. Case Presentation: A 34-week preterm female neonate (birth weight 1990 g, appropriate for gestational age) was born to a febrile primigravida with fetid greenish amniotic fluid at a regional secondary maternity and transferred at 30 h of life to our tertiary NICU with respiratory failure requiring mechanical ventilation. *L. monocytogenes* was recovered from blood, gastric aspirate, pharyngeal exudate, ocular secretion, and skin swab. Gradient strip susceptibility testing reported ampicillin and trimethoprim–sulfamethoxazole non-susceptibility, although confirmatory broth microdilution was unavailable. Broad-spectrum empirical therapy was revised on Day 5 to include ampicillin–sulbactam, with piperacillin–tazobactam and gentamicin continued. A follow-up blood culture on Day 9 remained sterile through 7 days. The hospital course was complicated by thrombocytopenia, transiently elevated aminotransferases, and a Grade I subependymal hemorrhage; tertiary NICU length of stay was 25 days. Conclusions: Recovery under a multi-agent regimen precludes attribution of effect to any single component. Discordant gradient strip susceptibility results in *L. monocytogenes* should be confirmed by broth microdilution before any therapeutic change; survivors of severe early-onset listeriosis require structured multidisciplinary follow-up.

## 1. Introduction

*Listeria monocytogenes* is a Gram-positive, facultative intracellular bacillus and the etiologic agent of human listeriosis, a severe foodborne disease that disproportionately affects pregnant women, neonates, the elderly, and immunocompromised hosts [1,2,3]. The pregnancy-associated form accounts for approximately 20–25% of all reported cases and is a leading cause of neonatal sepsis and meningitis of vertical origin [4,5,6,7]. In the European Union/European Economic Area, the notification rate of confirmed listeriosis has progressively increased over the past decade, reaching 2993 cases in 2023 (0.67 per 100,000 population) and 3041 confirmed invasive cases in 2024 (0.69 per 100,000), the highest annual figures since the inception of EU-wide surveillance; in 2024, listeriosis was also associated with the highest hospitalization and case-fatality proportions among all foodborne zoonoses reported in the EU [8,9]. Worldwide, estimates derived from the 2010 global burden of foodborne disease place the burden of listeriosis at approximately 23,150 illnesses and 5463 deaths, with perinatal cases accounting for a disproportionate share of severe outcomes [10].

Maternal–neonatal listeriosis presents in two clinical forms. Early-onset disease (≤6 days of life) typically results from transplacental dissemination during maternal bacteremia and is characterized by septicemia, respiratory failure, and granulomatosis infantiseptica. Late-onset disease (7–28 days) is more frequently meningeal and acquired through perinatal or nosocomial routes [5,11]. Hypervirulent clonal complexes, in particular CC1, CC2, CC4, and CC6, are over-represented in maternal–neonatal infection, reflecting their enhanced placental tropism mediated by internalin A (InlA) and InlB-dependent breaching of the syncytiotrophoblast barrier [6]. Historical series reported neonatal listeriosis mortality as high as 20–40% [12]. By contrast, the contemporary MONALISA prospective cohort of 189 liveborn neonates documented major adverse outcomes in 9% (3% deaths, 6% severe brain injury, 2% severe bronchopulmonary dysplasia), concentrated in infants born below 34 weeks of gestation [11]. Antenatal maternal antimicrobial therapy was associated with reduced neonatal severity at birth [11].

Treatment of neonatal listeriosis is based on intravenous ampicillin combined with an aminoglycoside (most commonly gentamicin) for synergistic bactericidal activity, with trimethoprim–sulfamethoxazole reserved as an alternative in patients with β-lactam allergy [13,14,15]. Acquired resistance in clinical *L. monocytogenes* isolates remains rare. In a French nationwide surveillance of 4816 strains, the prevalence of resistance was 1.27%, predominantly involving tetracyclines and fluoroquinolones, while resistance to first-line β-lactams was virtually absent [16]. A more recent French observational study of 2908 clinical isolates collected between 2012 and 2019, combined with 2431 food isolates from 2015 to 2019, confirmed 100% susceptibility to ampicillin and amoxicillin among clinical isolates [17]. Sporadic clinical isolates with reduced ampicillin susceptibility have nonetheless been described, and a *pbpB1* W428R substitution near the active site of penicillin-binding protein B1 has recently been associated with reduced β-lactam susceptibility, including cross-effects on amoxicillin and meropenem [18].

Despite the rarity of true ampicillin resistance, automated and gradient strip susceptibility methods may yield discordant results when applied to *Listeria*. Atypical gradient strip results should be confirmed by a reference minimum inhibitory concentration (MIC) method, particularly when they contradict the expected intrinsic susceptibility profile [19]. We report a 34-week preterm infant (birth weight 1990 g, appropriate for gestational age by weight, Fenton 2013 reference [20]) with multifocal early-onset neonatal listeriosis, in whom *L. monocytogenes* was recovered from blood and four peripheral sites, with gradient strip-reported ampicillin non-susceptibility. Molecular strain typing was not performed; therefore, clonal identity across sites cannot be confirmed. Clinical recovery and blood culture sterilization were achieved under combined antimicrobial therapy.

## 2. Case Presentation

This case report was prepared in accordance with the CARE guidelines [21]. All clinical events are reported with relative timing (Day 1 denotes the calendar day of birth; admission to our tertiary NICU occurred on Day 2 at approximately 30 h of life) and de-identified. The complete de-identified longitudinal laboratory, microbiological, cerebrospinal fluid, and TORCH serological dataset is provided in Supplementary Tables S2–S4.

### 2.1. Patient Information

A female preterm neonate of 34 weeks’ gestational age, born to a nulliparous primigravida (G1P0 at presentation; G1P1 following the index delivery) without documented antenatal care, was delivered by spontaneous vaginal delivery in cephalic presentation at a regional secondary maternity hospital. At delivery, the mother was febrile (the exact temperature was not recorded in the referring hospital’s transfer documentation), and the amniotic fluid was fetid and greenish, raising suspicion of clinical chorioamnionitis. The brief documented latency between rupture of membranes and delivery (≈10 min) is uncharacteristic of an ascending chorioamnionitic route and is more consistent with hematogenous transplacental seeding. Maternal dietary exposures associated with listeriosis (unpasteurized dairy, ready-to-eat deli meats, soft cheeses, smoked fish) were not retrievable from the referring hospital’s record. Maternal blood cultures, vaginal cultures, serological screening results, and placental histopathology were not available from the referring facility; chorioamnionitis and the route of vertical transmission could not therefore be microbiologically or histologically confirmed. The neonate was transferred at 30 h of life (Day 2) to the tertiary Neonatal Intensive Care Unit of “Cuza Vodă” Clinical Hospital of Obstetrics and Gynecology, Iaşi, Romania, where all subsequent care described in this report was delivered.

### 2.2. Clinical Findings

Anthropometric parameters at birth: weight 1990 g, appropriate for gestational age (between the 10th and 25th percentile for female infants at 34 weeks by the Fenton 2013 reference [20]); length and head circumference were not recorded at delivery. Apgar scores were 3 and 6 at 1 and 5 min, respectively. The neonate required immediate cardiorespiratory resuscitation, including positive-pressure ventilation with bag-and-mask, followed by early continuous positive airway pressure (CPAP). Initial physical examination revealed poor general condition, nasal flaring, bilateral subcostal retractions, expiratory grunting, axial hypotonia, and regular heart sounds without murmur.

Within hours of birth, the patient developed multiple apneic episodes, intermittent desaturations, and progressive respiratory deterioration requiring orotracheal intubation and mechanical ventilation. Blood group was determined as O Rh-negative. A packed red blood cell microtransfusion (30 mL, O Rh-negative) was administered on Day 1 at the referring facility for acute neonatal anemia in the context of severe early-onset sepsis with hemodynamic compromise. On admission to the tertiary NICU at 30 h of life (Day 2), the infant was febrile (axillary temperature 38.3 °C), with pink skin showing mild jaundice and scattered cutaneous ecchymoses, bilateral purulent ocular secretions, mechanical ventilation in pressure-controlled assist-control mode, thoracic bulging with bilateral intercostal retractions, harsh bilateral breath sounds, regular but tachycardic heart sounds, a soft non-tender abdomen, and a soft, non-bulging anterior fontanelle.

### 2.3. Timeline

**Figure 1:**
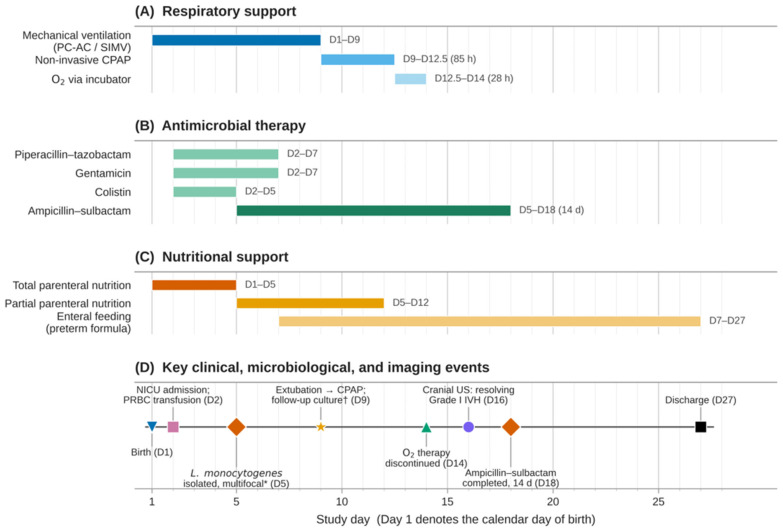
Integrated clinical course of the index neonate from birth (study day 1) to discharge (study day 27). Horizontal bars indicate the duration of (**A**) respiratory support, (**B**) antimicrobial therapy, and (**C**) nutritional support; the timeline in (**D**) marks key clinical, microbiological, and imaging events. Study day 1 denotes the calendar day of birth. * The isolate’s reported ampicillin non-susceptibility was determined by gradient-strip MIC testing and remained unconfirmed by broth microdilution† The follow-up blood culture obtained at extubation remained sterile through 7 days and was validated on day 16. Abbreviations: NICU, neonatal intensive care unit; PRBC, packed red blood cells; PC-AC, pressure-control assist-control; SIMV, synchronized intermittent mandatory ventilation; CPAP, continuous positive airway pressure; US, ultrasound; IVH, intraventricular haemorrhage.

A chronological summary of events is presented in Figure 1; the detailed day-by-day clinical timeline is provided in Supplementary Table S1.

### 2.4. Diagnostic Assessment

#### 2.4.1. Microbiology

*L. monocytogenes* was identified by Gram stain (short Gram-positive rods consistent with *Listeria* spp.). The initial blood culture smear additionally reported Gram-positive coccoid elements, which were not confirmed on subculture and were retrospectively interpreted as smear-reading artifacts. Further details are available in Supplementary Table S3. Species-level identification was performed using the VITEK^®^ 2 Compact automated identification system (bioMérieux, Marcy-l’Étoile, France) on isolates recovered from blood culture, gastric aspirate, pharyngeal exudate, ocular secretion, and skin swab, all collected on Day 2. The complete chronological microbiological assessment, including specimen type, method, culture result, and available susceptibility interpretation, is presented in Supplementary Table S3. The same species, *L. monocytogenes*, was recovered from all five sampled sites. An external-auditory-canal swab obtained concurrently grew only sparse polymorphic saprophytic Gram-positive flora, with *L. monocytogenes* not recovered; this sixth site is reported here to document pathogen-positive consecutive sampling rather than selective culture reporting. A concurrent fungal culture from the external auditory canal had been requested at the bedside and flagged for re-reading at 24–48 h; the definitive yeast culture result could not be retrieved from the laboratory information system at the time of manuscript preparation and is therefore acknowledged as an unresolved microbiological item rather than a documented negative finding.

Molecular strain typing was not performed; the manuscript therefore conservatively reports recovery of the same species rather than the same strain (Supplementary Table S3).

**Figure 2:**
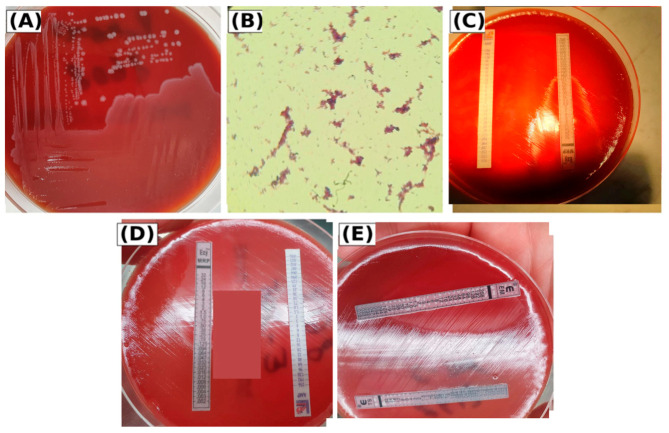
Microbiological documentation of *L. monocytogenes* recovered from the index blood culture isolate. (**A**) Subculture on sheep blood agar showing isolated grey-white pinpoint colonies. (**B**) Gram-stained smear from the primary blood culture broth (×1000, oil immersion); short Gram-positive rods consistent with *Listeria* spp. are visible alongside coccoid elements that were not confirmed on subculture and were retrospectively interpreted as smear-reading artifacts (Section 2.4.1; Supplementary Table S3). (**C**,**D**) Gradient strip MIC testing for ampicillin (AMP; Liofilchem MIC Test Strip, 256–0.016 µg/mL) and meropenem (MRP; Liofilchem *Ezy MIC*, 32–0.002 µg/mL) on the same plate, photographed under transmitted illumination (**C**) and, after repositioning of the plate, under reflected illumination (**D**). (**E**) Gradient strip MIC testing on a separate plate for erythromycin (EM; bioMérieux *Etest*, 256–0.016 µg/mL) and trimethoprim/sulfamethoxazole (TS; bioMérieux *Etest*, trimethoprim component 32–0.002 µg/mL, corresponding to sulfamethoxazole 608–0.038 µg/mL in the fixed 1:19 ratio). Numerical MIC readings could not be retrieved from the laboratory information system at the time of manuscript preparation. Only the categorical interpretation (resistant for AMP and TS; susceptible for MRP and EM) was retained and is presented here as gradient strip-reported and unconfirmed by broth microdilution. The solid red rectanglein panel (**D**) was added during figure preparation to redact handwritten patient-identifying annotations on the agar surface; all MIC gradient scales and antibiotic codes remain readable.

Antimicrobial susceptibility testing was performed on the blood culture and ocular-secretion isolates, with identical categorical interpretations: susceptible to meropenem and erythromycin, and resistant to ampicillin and trimethoprim–sulfamethoxazole by gradient strip MIC testing. Ampicillin and meropenem were tested with Liofilchem gradient strips (MIC Test Strip and *Ezy MIC*, respectively), and erythromycin and trimethoprim–sulfamethoxazole with bioMérieux *Etest* strips (Figure 2). For the gastric aspirate, pharyngeal exudate, and skin-swab isolates, only species-level identification was documented. The categorical susceptibility interpretation was the only output retrievable from the laboratory information system; numerical MIC readings could not be retrieved retrospectively, and the reported phenotype is therefore presented as gradient strip-reported and unconfirmed by broth microdilution. The European Committee on Antimicrobial Susceptibility Testing (EUCAST) does not currently endorse gradient strip methods as a reference technique for routine susceptibility testing of *Listeria monocytogenes*; broth microdilution against EUCAST breakpoints [19] remains the reference method and was not performed in this case.

Lumbar puncture was performed on Day 4, after approximately 48 h of broad-spectrum antimicrobial therapy, which reduces the sensitivity of CSF culture. The sample was hemorrhagic with clot formation, precluding reliable cytological analysis; bacterioscopy was negative and culture sterile. On bacterioscopy microscopy, frequent erythrocytes and relatively frequent polymorphonuclear leukocytes were noted (Supplementary Table S3); given the visibly hemorrhagic sample, this microscopic finding cannot be interpreted as a specific marker of meningeal inflammation. CSF biochemistry returned a glucose of 63 mg/dL, chloride 106 mmol/L, and protein 4.46 g/L (equivalent to approximately 446 mg/dL), the latter markedly above the gestational-age-adjusted reference range for preterm neonates (typically <1.5 g/L). In the context of a hemorrhagic lumbar tap, this elevated protein concentration cannot be interpreted as a specific marker of meningeal inflammation: the value is compatible both with blood–brain-barrier disruption associated with bacterial meningitis and with extensive blood contamination of the CSF sample, since visible blood at the time of puncture can elevate measured CSF total protein by several hundred mg/dL independently of any meningeal process. The CSF protein value was therefore documented but not used as a determinant of central nervous system involvement. Detailed CSF biochemical, cytological, bacterioscopic, and culture findings are reported in Supplementary Tables S2 and S3. Central nervous system involvement could not be definitively excluded despite sterile CSF culture. A single follow-up blood culture obtained on Day 9, during antimicrobial therapy, remained sterile at the 48 h preliminary reading and at the 7 d final reading on the automated continuous monitoring blood culture system, with definitive validation on Day 16; no further surveillance cultures were obtained, which is acknowledged as a limitation.

#### 2.4.2. Hematology and Biochemistry

**Figure 3:**
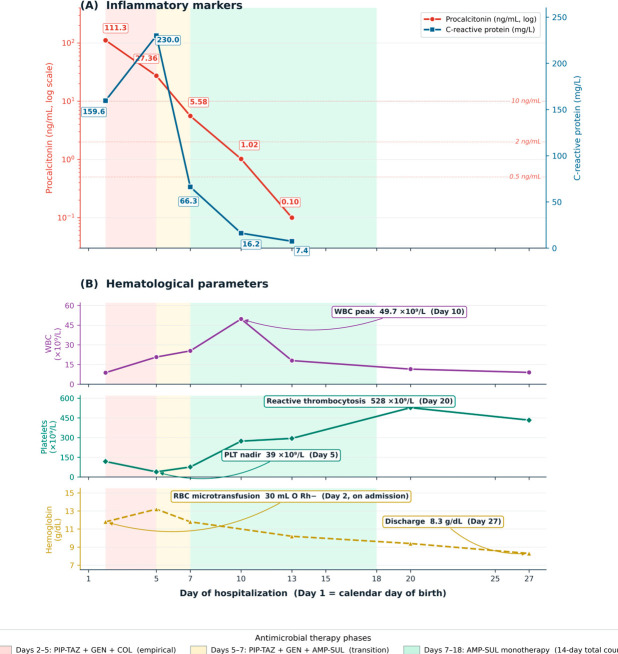
Longitudinal inflammatory and hematological profile during hospitalization. (**A**) Procalcitonin (PCT; left axis, log scale) and C-reactive protein (CRP; right axis, linear scale) from admission through Day 13. Dotted lines indicate the PCT reference thresholds of 0.5, 2, and 10 ng/mL; numeric annotations mark each measured value. (**B**) White blood cell count, platelet count, and hemoglobin displayed on a shared time axis in three stacked sub-panels, with key inflection points labeled (WBC peak, PLT nadir, reactive thrombocytosis, transfusion timing, discharge value). For clarity, the packed red blood cell microtransfusion was administered on Day 1 at the referring facility, prior to NICU admission; the figure annotation marks the hemoglobin value documented at NICU admission on Day 2. Background shading denotes the antimicrobial-regimen phases defined in the figure key. Day 1 denotes the calendar day of birth. AMP-SUL, ampicillin–sulbactam; COL, colistin; CRP, C-reactive protein; GEN, gentamicin; O Rh−, blood group O Rhesus-negative; PIP-TAZ, piperacillin–tazobactam; PLT, platelet count; RBC, red blood cell; WBC, white blood cell count.

On admission, the patient exhibited a marked acute-phase response (peak procalcitonin 111.3 ng/mL on Day 2; peak C-reactive protein 230.0 mg/L on Day 5), with inflammatory markers declining in parallel with clinical recovery (Figure 3A). The course was complicated by a verified thrombocytopenic nadir (39 × 10^9^/L on Day 5) followed by reactive thrombocytosis, a transient leukocytosis with marked left shift, transiently elevated aminotransferases, and self-limited electrolyte disturbances (hypokalemia, hypomagnesemia, and transient hyponatremia and hypercalcemia), all of which resolved during convalescence (Figure 3B). Anemia was present from admission and followed a convalescent decline to 8.3 g/dL at discharge; together with an inappropriately low Day-20 reticulocyte count, this pattern is consistent with combined anemia of prematurity and post-infectious hypoproliferative anemia. The transient Day-5 hemoglobin peak exceeded the surrounding values by more than the single 30 mL microtransfusion could explain and most likely reflects hemoconcentration at peak septic response. The complete time-resolved hematological, biochemical, coagulation, and inflammatory profile is provided in Supplementary Table S2.

#### 2.4.3. Imaging

Cranial ultrasonography on Day 4 described diffuse cerebral hypoechogenicity (operator’s verbatim wording), a non-specific pattern in this setting, compatible with hypoxic–ischemic encephalopathy (HIE), sepsis-associated cerebral edema, and/or early ischemic injury. Given the documented perinatal asphyxia (Apgar 3 at 1 min, requirement for positive-pressure ventilation followed by intubation), HIE represented a plausible contributor to this imaging pattern alongside sepsis-driven injury; therapeutic hypothermia was not initiated, since established cooling protocols are validated for infants ≥ 36 weeks’ gestation and evidence in the 34–35 week range remains limited. The absence of archived source images precluded retrospective re-review. Repeat cranial ultrasonography on Day 17 described a resolving right germinal-matrix/subependymal hemorrhage with cystic organization, compatible with prior Grade I hemorrhage. The administrative discharge code documented this finding as right intraventricular hemorrhage in remission, whereas the radiological description was of a subependymal/germinal-matrix hemorrhage (Papile Grade I); the manuscript adopts the imaging-based terminology throughout. Echocardiography on Day 9 showed a minor patent foramen ovale, Grade I tricuspid regurgitation, and a closed ductus arteriosus, all considered physiologic at this gestational age. Descriptions of cranial ultrasound findings are based on the operator’s written documentation in the medical record, because original images and formal radiology reports could not be retrieved from the institutional archive. Given the documented intracranial hemorrhage and prematurity, serial cranial ultrasound and neurodevelopmental follow-up were arranged after discharge.

#### 2.4.4. Differential Diagnosis

The initial differential diagnosis included early-onset sepsis caused by Group B *Streptococcus*, *Escherichia coli*, and other *Enterobacteriaceae*. Congenital cytomegalovirus, rubella, and toxoplasmosis were considered less likely because neonatal IgM testing was negative; CMV and rubella IgG positivity was interpreted as transplacentally acquired maternal antibody, while *Toxoplasma* IgG was below the assay positivity threshold. Maternal toxoplasmosis serological status was not available, so the neonatal result most likely reflects an immune-naive mother rather than excludes active fetal infection. The complete TORCH serological profile, including assay cut-offs and interpretation, is provided in Supplementary Table S4. Congenital CMV infection cannot be formally excluded retrospectively, because confirmatory CMV polymerase chain reaction testing on urine or saliva was not performed within the first 21 days of life. Disseminated neonatal herpes simplex virus (HSV) disease was a relevant competing diagnosis given the combination of respiratory failure, thrombocytopenia, elevated aminotransferases, and intracranial findings; HSV PCR and serology were not performed, and empirical acyclovir was not initiated. Recovery of *L. monocytogenes* from blood and multiple peripheral sites established the diagnosis of multifocal early-onset neonatal listeriosis, with probable vertical (transplacental and/or ascending) transmission.

### 2.5. Therapeutic Intervention

The empirical regimen at admission (detailed below) was broader than the ampicillin-plus-aminoglycoside combination conventionally recommended for early-onset neonatal sepsis [13,14]. The choice reflects the clinical context: an outborn neonate transferred at 30 h of life with established sepsis of uncertain etiology, in whom local protocols favored wide-spectrum Gram-positive and Gram-negative coverage pending pathogen identification. Although piperacillin–tazobactam has in vitro activity against *L. monocytogenes*, clinical equivalence to ampicillin in neonatal listeriosis is not established. Colistin was added empirically in view of institutional concern regarding multidrug-resistant Gram-negative pathogens in neonates transferred from external units and was discontinued promptly once *L. monocytogenes* was identified and no multidrug-resistant Gram-negative organism was recovered. Its inclusion reflected empirical uncertainty at admission rather than a guideline-based recommendation and added limited value to the management of this Gram-positive infection. The case nonetheless illustrates the value of including ampicillin in initial empirical coverage whenever *L. monocytogenes* is clinically plausible, most clearly in any preterm infant born to a febrile mother with fetid amniotic fluid, while recognizing that broader empirical regimens may be reasonably selected in transferred patients with undifferentiated sepsis pending pathogen identification.

The empirical regimen administered from Day 2 comprised intravenous piperacillin–tazobactam at 80 mg/kg/dose (piperacillin component) every 6 h (corresponding to 160 mg piperacillin plus 20 mg tazobactam per administration in this 1990 g infant), gentamicin at 5 mg/kg/dose every 36 h according to an extended-interval regimen appropriate for postmenstrual age 30–35 weeks (10 mg per dose), and colistin administered as colistimethate sodium at 1.7 mg/kg/dose of colistin base activity every 8 h (3.4 mg of colistin base activity per dose, equivalent to approximately 42,500 IU). On Day 5, following microbiological identification, colistin was discontinued and ampicillin–sulbactam (2:1) was added at an ampicillin dose of 50 mg/kg every 12 h (100 mg ampicillin plus 50 mg sulbactam per administration), increased to 75 mg/kg every 12 h (150 mg ampicillin plus 75 mg sulbactam per administration) once postnatal age exceeded 7 days, in accordance with the gestational- and postnatal-age-stratified ampicillin pharmacokinetic regimen. Piperacillin–tazobactam and gentamicin were continued for two further days as bridging cover and were discontinued on Day 7. Ampicillin–sulbactam was then maintained as the sole antimicrobial agent to complete a total course of 14 days, with the final dose administered on Day 18. Because two β-lactam/β-lactamase-inhibitor combinations (piperacillin–tazobactam and ampicillin–sulbactam) and an aminoglycoside (gentamicin) overlapped in time, the contribution of any single component to the observed clinical recovery cannot be isolated. Therapeutic drug monitoring of plasma gentamicin and colistimethate concentrations was not available at our institution and was therefore not performed. Routine clinical and laboratory surveillance during therapy comprised serum creatinine, urine output, full blood count, and hepatic transaminases, none of which deviated from age-expected reference ranges, and no clinically apparent drug-related adverse events were recorded.

The use of ampicillin–sulbactam should be interpreted pragmatically rather than mechanistically: in the absence of β-lactamase testing or resistance-gene characterization, no conclusion can be drawn regarding a specific contribution of sulbactam. The decision was driven by the well-documented intrinsic susceptibility of *L. monocytogenes* to β-lactams and by the possibility of discordant gradient strip results in slow-growing organisms. Current expert recommendations support at least 14 days of effective antimicrobial therapy for neonatal listeriosis with documented bacteremia in the absence of proven central nervous system involvement, and at least 21 days when meningitis is established or strongly suspected, based on the susceptibility of *L. monocytogenes* to intracellular persistence and on the recognized risk of relapse after shorter courses [14,15]. A 14-day course was selected based on microbiologically confirmed bacteremia with sterile follow-up blood culture, resolution of inflammatory markers, and absence of clinical neurological deterioration, consistent with Red Book guidance for *Listeria* bacteremia without proven meningitis [14]. A 21-day meningitis-directed course was considered but was not adopted given the negative bacterioscopy and culture on the hemorrhagic CSF sample.

The infant was intubated within hours of birth at the referring hospital and remained on invasive mechanical ventilation until extubation on Day 9; supportive modes comprised pressure-control assist-control for 4 days, synchronized intermittent mandatory ventilation for 48 h, and endotracheal CPAP for 3 h, followed by non-invasive nasal CPAP for 85 h and supplemental oxygen via incubator for 28 h, with oxygen discontinued on Day 14. Nutritional management comprised total parenteral nutrition for the first 5 days (Days 1–5) and partial parenteral nutrition for 7 days, with progressive enteral feeding by orogastric gavage with preterm formula introduced from Day 7 onward and advanced according to digestive tolerance. Adjunctive interventions included intramuscular vitamin K_1_ prophylaxis, packed red blood cell microtransfusion (30 mL, O Rh-negative) on Day 1 at the referring facility, oral iron supplementation from Day 22, vitamin D_3_ from Day 13, probiotics, acid–base and electrolyte correction, thermoregulation, and continuous monitoring of vital functions.

### 2.6. Follow-Up and Outcomes

Clinical evolution was slowly favorable. The patient was extubated on Day 9, weaned from non-invasive support by Day 14, and achieved full enteral feeding with progressive weight gain prior to discharge. On Day 27 (postmenstrual age 38 weeks), the infant was transferred back to the referring hospital for continued convalescent care. At transfer, she was clinically stable, afebrile, with normal cardiopulmonary examination, soft non-tender abdomen, full enteral tolerance (50 mL × 8 feeds per day), and normal stools and urinary output. The tertiary NICU length of stay was 25 days (admission Day 2 to discharge Day 27); chronological age at discharge was 27 days, corresponding to a postmenstrual age of 38 weeks. Anthropometrics at discharge: weight 2440 g, length 48 cm, head circumference 32.5 cm.

The infant survived to discharge with clinical stabilization, full enteral feeding, and improving inflammatory and hematological parameters, but with persistent anemia (hemoglobin 8.3 g/dL at discharge, declining progressively from a transient Day-5 peak of 13.2 g/dL through 11.8, 10.2, and 9.4 g/dL on Days 7, 13, and 20). As discussed in Section 2.4.2, the Day-5 peak most likely reflects hemoconcentration rather than a delayed effect of the single Day-1 microtransfusion. The overall pattern, together with the inappropriately low Day-20 reticulocyte count (6/1000; 0.6%), is consistent with combined anemia of prematurity and post-infectious hypoproliferative anemia; oral iron supplementation was initiated from Day 22. Newborn hearing screening using transient evoked otoacoustic emissions (TEOAE) recorded a bilateral REFER result. Diagnostic auditory brainstem response (ABR) testing was scheduled for outpatient follow-up at 3 months of corrected age. In the context of culture-proven invasive listeriosis, which is a recognized cause of sensorineural hearing loss, an earlier confirmatory ABR would have strengthened the audiological pathway, and the present case has informed an ongoing institutional review of pre-discharge audiological assessment in this population. Phenylketonuria, congenital hypothyroidism, and cystic fibrosis screenings were sent and pending at discharge. A structured neurodevelopmental, audiological, ophthalmological, and cardiological follow-up plan was arranged at discharge.

Follow-up was organized across the high-risk newborn clinic of “Cuza Vodă” Clinical Hospital of Obstetrics and Gynecology and the regional pediatric specialty services at Emergency Children’s Hospital, and included high-risk neurodevelopmental surveillance at 3 and 6 months corrected age (addressing the documented Grade I subependymal hemorrhage and the differential consideration of HIE); diagnostic ABR audiological reassessment at 3 months in view of the bilateral OAE REFER and the recognized SNHL risk after invasive listeriosis; ophthalmologic screening for retinopathy of prematurity within 4–6 weeks; cardiology follow-up at 3 months to document closure of the patent foramen ovale; inclusion in the palivizumab prophylaxis program for respiratory syncytial virus during October–March; and routine immunization adjusted to chronological age. Bacillus Calmette–Guérin (BCG) vaccination was deferred until clinical stabilization criteria were met, with administration scheduled at the first scheduled outpatient review.

### 2.7. Patient Perspective

The mother was informed in detail about the nature of the infection, the likely modes of transmission of *Listeria monocytogenes* in pregnancy (predominantly through ingestion of contaminated foods, although a specific source could not be retrospectively identified), the necessity of antenatal medical surveillance, and the long-term neurodevelopmental follow-up. At initial disclosure of the microbiological diagnosis, the mother reported anxiety about possible dietary causes and asked whether her food choices during pregnancy might have been responsible for the infection. The clinical team explained that listeriosis in pregnancy is acquired predominantly through ingestion of contaminated foods, that no specific dietary source could be identified retrospectively in this case, and that such exposures are common and frequently unavoidable; the mother was reassured that the infection did not reflect personal fault, while being counseled that awareness of high-risk foods and antenatal surveillance can reduce the risk in any subsequent pregnancy. She engaged actively with the discharge education program, including written information on listeriosis-associated food risks, and confirmed her understanding of why each scheduled developmental, audiological, ophthalmological, and cardiological follow-up appointment was being arranged. She expressed gratitude for the multidisciplinary care and committed to scheduled follow-up visits and preventive counseling for any subsequent pregnancy.

## 3. Discussion

This report illustrates several clinical and microbiological challenges in neonatal listeriosis. It documents multifocal early-onset neonatal listeriosis in a preterm infant born appropriate for gestational age, following suspected clinical chorioamnionitis. It also documents an unexpected gradient strip-reported ampicillin non-susceptibility result that was not confirmed by broth microdilution, together with favorable short-term clinical and microbiological evolution under combined antimicrobial therapy.

### 3.1. Multifocal Isolation in Maternal–Fetal Listeriosis

Recovery of *L. monocytogenes* from blood, gastric aspirate, pharyngeal exudate, ocular secretion, and skin swabs is most consistent with extensive transplacental and amniotic-fluid–mediated dissemination, consistent with the granulomatosis infantiseptica spectrum described in classical accounts of neonatal listeriosis [22]. In the MONALISA cohort, *L. monocytogenes* was recovered from systemic samples (blood, CSF, or urine) in nearly all neonates with maternal–fetal listeriosis, and 70% had abnormal clinical status at birth, including respiratory distress in 56% [11], a phenotype closely matching the patient described here. Multifocal peripheral isolation, although less frequently emphasized in published series, supports the hypothesis of late intrauterine bacterial showering, often preceded by a maternal flu-like illness one to two weeks before delivery [5,6,11,23]. The absence of antenatal care in this case precluded detection of the typical maternal prodrome. NICU-acquired cross-infection between neonates [24] was considered unlikely here: no other concurrent case of listeriosis was identified in the unit during the admission, no shared incubator or respiratory-circuit exposure was documented, and microbiological recovery occurred from samples obtained within hours of arrival at the tertiary center.

A terminological clarification is warranted. In the absence of molecular strain typing (pulsed-field gel electrophoresis or whole-genome sequencing), the term “multifocal” is used in this manuscript in the strictly anatomical sense, to denote recovery of *Listeria monocytogenes* from five anatomically distinct sites, rather than to imply a single proven clone. Although the clinical pattern of simultaneous recovery from blood and several peripheral sites is most parsimoniously explained by intrauterine dissemination of a single clone, this assumption cannot be formally substantiated and is acknowledged as a limitation. The observed phenotype is compatible with the established pathophysiology of maternal–fetal listeriosis involving placental invasion and hematogenous fetal dissemination. Hypervirulent clonal complexes (CC1, CC2, CC4, and CC6) are over-represented in maternal–neonatal disease and would have been the natural target of clonal-complex assignment. However, because viable isolates were not retained after routine clinical processing, retrospective molecular characterization is no longer possible.

### 3.2. Discordant Antibiogram and Methodologic Considerations

The defining feature of this case is the reported in vitro non-susceptibility to ampicillin by gradient strip testing, which is discordant with the expected susceptibility profile of *L. monocytogenes* to aminopenicillins (Figure 2). In a nationwide French analysis of 4816 clinical *L. monocytogenes* strains collected since 1926, only 1.27% of isolates exhibited acquired antimicrobial resistance, and resistance to first-line β-lactams was virtually absent [16]. A more recent observational study of 2908 clinical isolates collected in France between 2012 and 2019 confirmed 100% susceptibility of clinical isolates to ampicillin and amoxicillin [17], reinforcing the rarity of true acquired β-lactam resistance in this species. In an independent Swiss survey of foodborne, clinical, and environmental *Listeria* spp. isolates, a small minority of strains were observed near the intermediate or resistant ampicillin breakpoint, although confirmation of acquired β-lactam resistance was not established [25].

More than one explanation can account for the present observation. *L. monocytogenes* is intrinsically resistant to fosfomycin and to all third- and fourth-generation cephalosporins; the species-level breakpoint for ampicillin (MIC ≤ 1 mg/L for susceptibility per EUCAST) is based on broth microdilution and may yield discordant results when extrapolated to gradient strip methods in slow-growing isolates [19,25]. Rare mechanisms of reduced β-lactam susceptibility have been described in *L. monocytogenes*, most recently a *pbpB1* W428R substitution near the active site of penicillin-binding protein B1, which confers cross-effects on amoxicillin and meropenem [18]. The reported meropenem susceptibility in our isolate makes this mechanism an unlikely explanation, since *pbpB1* W428R typically elevates meropenem MICs in parallel with ampicillin; the discordant result is therefore more plausibly methodological. Confirmatory broth microdilution with EUCAST breakpoints was not available in our laboratory, where gradient strip MIC testing is the routine method; its absence reflects a standing limitation of the service rather than an omission specific to this case, and it remains the central methodologic limitation of the report. Targeted genomic interrogation of *pbpB1* and a phenotypic β-lactamase assay (e.g., nitrocefin) were not feasible retrospectively at our institution, and the mechanistic basis of the discordant phenotype therefore remains undetermined.

Given these considerations, the gradient strip result is unexpected for *L. monocytogenes* and should be regarded as unconfirmed in the absence of broth microdilution. The clinical response observed under the combined regimen of piperacillin–tazobactam, gentamicin, and ampicillin–sulbactam, documented by sterilization of blood cultures at 48 h and 7 days, by progressive normalization of inflammatory markers (Figure 3), and by clinical recovery, is compatible with the hypothesis that the reported in vitro ampicillin non-susceptibility did not translate into therapeutic failure. We do not propose ampicillin–sulbactam as salvage therapy for neonatal listeriosis. Recovery occurred under combined β-lactam and aminoglycoside coverage, and the individual contribution of any single agent cannot be isolated from this single observation. Sulbactam is a mechanism-based (“suicide”) β-lactamase inhibitor with negligible intrinsic antibacterial activity, except against *Acinetobacter baumannii* and *Neisseria gonorrhoeae*; it would have contributed to the clinical effect only if a β-lactamase-mediated mechanism were involved [26], a hypothesis that remains unverified in the absence of resistance-gene characterization or β-lactamase assay.

The same isolate was categorized as resistant to trimethoprim–sulfamethoxazole by gradient strip testing. This result is likewise discordant with the expected profile of *L. monocytogenes*, which is characteristically susceptible to trimethoprim–sulfamethoxazole, the recommended alternative agent in β-lactam allergy [13,14,15]. Gradient strip determination of trimethoprim–sulfamethoxazole minimum inhibitory concentrations is additionally susceptible to trailing endpoints and inter-reader variability in fastidious organisms, and was not confirmed by broth microdilution against EUCAST breakpoints [19]. As for ampicillin, the trimethoprim–sulfamethoxazole result should therefore be regarded as gradient strip-reported and unconfirmed; it did not affect first-line management, which appropriately relied on a β-lactam plus an aminoglycoside.

### 3.3. Comparison with Published Cases

Table 1 summarizes a narratively selected (non-systematic) sample of published cases and series of early-onset neonatal listeriosis with documented multifocal isolation or atypical antimicrobial findings. The patient described in this report is distinguished by the combination of multifocal microbiological recovery, reported in vitro β-lactam non-susceptibility, and favorable short-term clinical evolution under a regimen that included a β-lactam/β-lactamase inhibitor combination.

Reports published since the MONALISA studies, summarized in Table 1, span the full clinical spectrum of neonatal listeriosis: respiratory-predominant disease in preterm infants treated successfully with ampicillin-based regimens [27]; severe central nervous system involvement with variable neurodevelopmental outcomes [28,29]; and fulminant, rapidly fatal courses despite appropriate therapy [30,31]. Long-term follow-up further indicates an increased risk of executive, motor, and cognitive impairment at 5 years, attributable largely to prematurity rather than to infection per se [32]. The patient described here adds to this literature by documenting a discordant in vitro susceptibility result coupled with multifocal microbiological recovery and short-term clinical recovery under a β-lactam/β-lactamase inhibitor combination.

### 3.4. Clinical Implications

In any preterm neonate born to a febrile mother with fetid amniotic fluid, *L. monocytogenes* should be considered alongside Group B *Streptococcus* and *Escherichia coli* as a probable etiologic agent, even in the absence of confirmed maternal exposure [11,13,23]. Empirical treatment for early-onset neonatal sepsis should therefore include ampicillin whenever *L. monocytogenes* is clinically plausible, especially in preterm infants born to febrile mothers with foul-smelling amniotic fluid.

Multifocal screening, encompassing blood, gastric aspirate, pharyngeal, conjunctival, and skin swabs at admission, may improve pathogen recovery, particularly when CSF sampling is technically compromised, as in the present case. When atypical antimicrobial resistance is reported in an organism with well-defined intrinsic susceptibility, confirmation by an alternative reference method (broth microdilution) and consultation of the National Reference Center are warranted before any therapeutic de-escalation [19,25].

A practical aspect highlighted by this case was the rapid microbiological identification of *Listeria monocytogenes*. Following isolation in pure culture, species identification using the VITEK^®^ 2 Compact system (bioMérieux, Marcy-l’Étoile, France) was achieved within approximately 6 h, facilitating early therapeutic reassessment in a critically ill neonate. Subsequent acquisition in our laboratory of MALDI-TOF MS technology using the VITEK^®^ MS PRIME system (bioMérieux) has further reduced identification time to seconds from pure colonies, which is expected to improve diagnostic timeliness in future similar cases.

Antenatal counseling regarding listeriosis-associated dietary risks (unpasteurized dairy, ready-to-eat deli meats, smoked fish, soft cheeses), combined with structured antenatal surveillance for under-supervised pregnancies, remains an actionable preventive strategy in the perinatal setting [20].

### 3.5. Strengths and Limitations

Strengths of this report include multi-site microbiological documentation, longitudinal tracking of inflammatory markers, structured neuroimaging follow-up, and a transparent chronological account of therapeutic decision-making in a real-world clinical setting. Several limitations qualify the interpretation of the findings and are presented here grouped by theme rather than enumerated.

Microbiological methodology. Antimicrobial susceptibility was characterized by gradient strip MIC testing, without confirmatory broth microdilution. Confirmatory broth microdilution, serotyping, and whole-genome sequencing (WGS) could not be performed retrospectively because viable isolates were not retained after routine clinical processing. Numerical MIC values could not be retrieved retrospectively from the laboratory information system, although the identification platform (VITEK^®^ 2 Compact, bioMérieux) and the gradient strip products used (Liofilchem for ampicillin and meropenem; bioMérieux Etest for erythromycin and trimethoprim–sulfamethoxazole) have been confirmed. The test-execution parameters of the institutional standard protocol (blood agar, an inoculum of approximately 10^8^ CFU/mL suspension density equivalent to a 0.5 McFarland standard, aerobiosis, and 18–25 h incubation) were applied. The reported ampicillin and trimethoprim–sulfamethoxazole non-susceptibility phenotype is therefore best interpreted as gradient strip-reported and unconfirmed rather than as established acquired resistance. Accordingly, the manuscript describes recovery of the same species at five sites rather than clonal identity. Serotyping and clonal-complex assignment (CC1/CC2/CC4/CC6) were not performed, and the placental-tropism mechanisms outlined in the Introduction cannot be linked to the present isolate.

Maternal and placental workup. Maternal blood cultures, vaginal cultures, and placental histopathology were not available from the referring facility, since the neonate was transferred at 30 h of life and the mother did not re-attend and could not be contacted after the infant’s discharge, so the maternal microbiological and symptomatic profile beyond intrapartum fever could not be retrospectively characterized. Vertical transmission and clinical chorioamnionitis therefore remain probable rather than formally established. In the absence of placental polymerase chain reaction testing, the route of vertical transmission cannot be discriminated between transplacental hematogenous seeding and an ascending intrapartum route, although the very short documented interval between rupture of membranes and delivery (≈10 min) favors the former.

Central nervous system workup. Lumbar puncture was performed after approximately 48 h of empirical antimicrobial therapy, and the CSF sample was hemorrhagic, limiting both cytological assessment and culture sensitivity for meningeal involvement. A pre-treatment lumbar puncture would have strengthened the CNS workup, and the case has informed an internal review of admission diagnostic pathways for suspected early-onset bacterial meningitis. A repeat lumbar puncture at 48–72 h or after clinical improvement was not performed; the decision at the time was supported by sustained clinical improvement, defervescence, and progressive normalization of inflammatory markers. Disseminated neonatal herpes simplex virus (HSV) disease was considered clinically unlikely given the overall presentation and microbiological findings and was excluded on clinical grounds; HSV PCR and serology were not obtained, and empirical acyclovir was not initiated. Original cranial ultrasound images could not be retrieved at the time of manuscript preparation, and imaging descriptions are therefore based on the operator’s written documentation. The Day-4 finding of diffuse cerebral hypoechogenicity is a non-specific imaging pattern compatible with hypoxic–ischemic encephalopathy (HIE), sepsis-associated cerebral edema, ischemic injury, or early hemorrhagic infiltration; HIE was not formally diagnosed or excluded, and the relative contribution of perinatal asphyxia versus sepsis-driven injury to the eventual subependymal hemorrhage cannot be disaggregated retrospectively. The absence of source images precluded retrospective expert reinterpretation. Congenital cytomegalovirus was considered unlikely on the basis of negative neonatal IgM and transplacentally acquired IgG, although neonatal urine or saliva CMV polymerase chain reaction was not performed within the 21-day diagnostic window and is no longer back-dateable.

Treatment monitoring and pharmacological documentation. A single follow-up blood culture (Day 9) was obtained to document microbiological response under antimicrobial therapy; a single negative culture during ongoing therapy is recognized as relatively weak evidence of microbiological cure in invasive listeriosis, in which intracellular bacterial persistence is biologically plausible. Weight-adjusted antimicrobial doses are reported in Section 2.5. Therapeutic drug monitoring of plasma gentamicin and colistimethate concentrations was not available at our institution and was therefore not performed, with aminoglycoside dosing relying on validated postmenstrual-age-stratified extended-interval regimens. The favorable clinical and microbiological outcome, together with the absence of clinically apparent nephrotoxicity or ototoxicity at discharge, is consistent with adequate dosing in this individual patient, although this observation cannot be generalized. As discussed in Section 2.5, the initial empirical regimen did not include ampicillin, which is the agent specifically recommended in international guidelines when *L. monocytogenes* is a plausible pathogen; in light of the present experience, the unit has incorporated ampicillin into the empirical regimen for transferred neonates with comparable maternal risk factors.

Follow-up and literature comparison. Follow-up was limited to the immediate post-discharge period, and longer-term hematological and neurodevelopmental outcomes are not yet available, although structured multidisciplinary outpatient surveillance was arranged at discharge. Newborn hearing screening using TEOAE recorded a bilateral REFER result, and confirmatory ABR testing was scheduled for outpatient reassessment at 3 months of corrected age. Given that culture-proven invasive listeriosis is a recognized cause of sensorineural hearing loss, earlier confirmatory ABR would have strengthened the audiological pathway; this observation has prompted an institutional review of pre-discharge audiological assessment for neonates with invasive bacterial infection. The accompanying fungal culture from the external auditory canal, flagged at the bedside for re-reading at 24–48 h, could not be retrieved from the laboratory information system retrospectively. The literature comparison in Table 1 was assembled by narrative selection rather than by systematic search.

As with all single-patient observations, the present report cannot establish a causal contribution of ampicillin–sulbactam to the favorable short-term outcome, particularly given the concomitant administration of piperacillin–tazobactam and gentamicin; the findings are hypothesis-generating and warrant confirmation in larger, prospectively collected series. Despite these limitations, the report offers a transparent and longitudinally documented account of multifocal early-onset neonatal listeriosis in a preterm infant with a discordant gradient strip-reported susceptibility profile. The case adds to the limited published experience on this rare presentation and reinforces practical lessons concerning the need for confirmatory broth microdilution before any therapeutic change, the value of multi-site microbiological documentation, and the importance of structured multidisciplinary follow-up after invasive neonatal listeriosis.

### 3.6. Take-Home Messages

• Multifocal early-onset neonatal listeriosis should be considered in preterm infants born to febrile mothers with foul-smelling, meconium-stained, or fetid amniotic fluid, especially when antenatal care was absent.
• Recovery of *L. monocytogenes* from multiple sites supports extensive fetal/neonatal exposure, but molecular typing is required before claiming clonal identity.
• Gradient strip-reported ampicillin non-susceptibility in *L. monocytogenes* should be confirmed by broth microdilution before any change to standard therapy.
• Empirical treatment for early-onset neonatal sepsis should include ampicillin when *L. monocytogenes* is clinically plausible.
• Survivors of invasive neonatal listeriosis require structured neurodevelopmental, audiological, ophthalmological, and hematological follow-up; given the recognized risk of sensorineural hearing loss after this pathogen, otoacoustic emission screening should be supplemented by diagnostic ABR irrespective of the initial OAE result.

## 4. Conclusions

In *Listeria monocytogenes*, a species with well-established intrinsic susceptibility to first-line β-lactams, a discordant gradient strip non-susceptibility phenotype should be confirmed by broth microdilution against EUCAST breakpoints, with consultation of the National Reference Center, before any therapeutic change. Multifocal early-onset neonatal listeriosis in a 34-week preterm infant was documented here, with *L. monocytogenes* recovered from blood and four peripheral sites and with a gradient strip-reported but broth-microdilution-unconfirmed ampicillin non-susceptibility phenotype. Sterile follow-up blood culture and short-term clinical recovery occurred under a combined regimen of ampicillin–sulbactam, piperacillin–tazobactam, and gentamicin; because these agents overlapped temporally, the contribution of any single component cannot be isolated, and the case does not support deviation from the standard ampicillin-plus-aminoglycoside regimen. Empirical coverage of early-onset neonatal sepsis in any preterm infant born to a febrile mother with fetid amniotic fluid should include ampicillin, and survivors of invasive early-onset neonatal listeriosis require structured multidisciplinary follow-up that incorporates diagnostic ABR irrespective of the initial OAE outcome. Finally, antenatal counseling on listeriosis-associated dietary risks, together with structured antenatal surveillance for under-supervised pregnancies, remains an actionable preventive priority in the perinatal setting.

## Figures and Tables

**Table 1:** Selected literature contextualizing neonatal listeriosis and antimicrobial susceptibility findings in *Listeria monocytogenes*, with positioning of the present report. The literature comparison was assembled by narrative selection rather than systematic search, BW, birth weight; CNS, central nervous system; EOL, early-onset listeriosis; GA, gestational age; LOL, late-onset listeriosis; MIC, minimum inhibitory concentration; NICU, neonatal intensive care unit; WGS, whole-genome sequencing.

Study (Year)	Design/Setting	Population/Material	Relevance to Present Case	Main Finding
Charlier et al., 2017 [4]	Prospective national cohort (MONALISA), France (2009–2013)	818 listeriosis cases from 372 centers (107 maternal–neonatal, 427 bacteremia, 252 neurolisteriosis)	Reference cohort for clinical features and predictors of mortality and persisting impairment	Severity of listeriosis higher than reported elsewhere; identified neurolisteriosis dexamethasone-associated reduced survival and defined the time-window for fetal loss
Charlier et al., 2022 [11]	Prospective national cohort (Neonatal MONALISA), France (2009–2017)	189 liveborn neonates with maternal–neonatal listeriosis (132 EOL, 12 LOL; remainder without active neonatal listeriosis)	Benchmark for contemporary neonatal listeriosis severity and outcome	70% abnormal status at birth (133/189); 56% respiratory distress (106/189); 22% extreme/very preterm; major adverse outcomes in 9–3% deaths (5/189), 6% severe brain injury (12/189), 2% severe bronchopulmonary dysplasia (3/189), concentrated in infants < 34 weeks GA
Morvan et al., 2010 [16]	National surveillance, France (strains 1926–2007)	4816 clinical *L. monocytogenes* isolates from the French National Reference Center	Background prevalence of acquired antimicrobial resistance in clinical isolates	Acquired resistance in 1.27% of clinical isolates, predominantly to tetracyclines and fluoroquinolones; clinically relevant antibiotics (β-lactams, aminoglycosides) remained generally effective
Moura et al., 2024 [17]	Observational study, France (clinical isolates 2012–2019; food isolates 2015–2019)	2908 clinical and 2431 food *L. monocytogenes* isolates with phenotypic susceptibility testing and whole-genome sequencing	Contemporary confirmation that acquired β-lactam resistance in clinical isolates remains exceptional	All clinical isolates susceptible to ampicillin and amoxicillin; acquired resistance in 0.98% of clinical and 3.66% of food isolates, predominantly affecting tetracyclines
Bertsch et al., 2014 [25]	Surveillance study, Switzerland (food, clinical, environmental)	524 *Listeria* spp. isolates (155 of human clinical origin)	Documents methodologic discordance near the ampicillin breakpoint and confirms that ampicillin intermediate-zone MICs occur in the absence of acquired resistance genes	All isolates susceptible to amoxicillin and penicillin; ampicillin intermediate-zone MICs (up to 4 µg/mL) observed without detection of acquired ampicillin-resistance genes; overall acquired resistance 16/524 (3.1%), 92/524 (17.6%) intermediate to at least one antimicrobial
Wamp et al., 2026 [18]	Single clinical index isolate (Germany, 2023) with retrospective WGS database screen and in vitro selection experiments	One index isolate + 8 additional *pbpB1* W428R-positive clinical isolates + EGD-e suppressor mutants	Mechanism of reduced β-lactam susceptibility in *L. monocytogenes*; β-lactam cross-resistance directly relevant to the present case	A *pbpB1* W428R substitution near the active site of penicillin-binding protein B1 reduces susceptibility to ampicillin, amoxicillin, and meropenem; the same mutation was experimentally selected under both ampicillin and meropenem exposure, indicating cross-selectability across β-lactam classes
Zhang et al., 2023 [27]	Retrospective single-center series, China (2015–2022)	11 neonates with culture-confirmed listeriosis	Contemporary cohort with predominantly respiratory presentation, comparable to the present case	Respiratory distress in 100%; majority preterm; favorable response to ampicillin-based regimens
Maddaloni et al., 2026 [28]	Retrospective tertiary-NICU case series, Italy	4 neonates with central nervous system involvement	Spectrum of CNS sequelae in EOL despite microbiologically appropriate therapy	Severe EOL complicated by ventriculitis, hydrocephalus, and seizures; outcomes ranged from near-normal neurodevelopment to profound impairment
Rodrigues Amaral et al., 2025 [29]	Single case report, Portugal	1 preterm neonate with severe EOL	Diagnostic-challenge context analogous to the present case	Reinforces the diagnostic value of placental polymerase chain reaction testing as a complementary modality when clinical microbiology is delayed
Simão Raimundo et al., 2023 [30]	Single case report, Portugal (Azores)	1 male preterm neonate, 34 weeks GA, meconium-stained amniotic fluid	Closely matched phenotype: 34-week gestational age, meconium-stained amniotic fluid, fetal distress, EOL with septic shock	*L. monocytogenes* recovered from blood with negative CSF and urine cultures; required 10 days of mechanical ventilation and high-dose long-term antimicrobial therapy; favorable outcome
Tao et al., 2025 [31]	Single case report, China	1 preterm female neonate, 33 + 5 weeks GA	Illustrates the fulminant pole of the EOL spectrum and the lethal potential despite microbiologically appropriate therapy	Fulminant EOL with septic shock, capillary leak syndrome, and multi-organ failure; mortality within 24 h despite penicillin plus meropenem
Charlier et al., 2023 [32]	Prospective matched observational cohort, France (MONALISA follow-up)	Surviving children with neonatal listeriosis assessed at 5 years against matched controls	Provides the long-term neurodevelopmental rationale for structured multidisciplinary follow-up after neonatal listeriosis	Long-term outcomes mainly attributable to prematurity rather than infection per se; persistent executive-function and motor sequelae documented at age 5; supports systematic long-term screening
Present case	Single-patient case report, Romania	1 preterm neonate (34 weeks, BW 1990 g)	Index case for multifocal EOL with gradient strip-reported, broth-microdilution-unconfirmed ampicillin and trimethoprim–sulfamethoxazole non-susceptibility	Sterile follow-up blood culture and short-term clinical recovery under combined antimicrobial therapy (piperacillin–tazobactam + gentamicin + ampicillin–sulbactam); subependymal/germinal-matrix hemorrhage; reactive thrombocytosis; persistent anemia at discharge

## Data Availability

The de-identified clinical, laboratory, microbiological, and imaging data summarized in this case report are presented within the article and accompanying tables and figures. The complete primary medical record is not publicly available because of patient-privacy restrictions and the increased risk of re-identification inherent in a single rare pediatric case from a single named center. Specific de-identified source items may be made available from the corresponding author upon reasonable request, where available and subject to ethics-committee re-evaluation. Source identifiers (patient name, parental contact details, hospital case number, attending physicians’ names) have been removed from all materials prior to any external sharing.

## Supplementary Materials

The following supporting information can be downloaded at: https://www.mdpi.com/article/10.3390/pathogens15070674/s1, Table S1: Chronological timeline of clinical events; Table S2: Longitudinal hematological, biochemical, coagulation, inflammatory marker, and cerebrospinal fluid profile; Table S3: Longitudinal microbiological assessment during hospitalization; Table S4: TORCH serological profile; supplementary CARE 2017 reporting checklist.

## Abbreviations

ABR, auditory brainstem response; AMP-SUL, ampicillin–sulbactam; BCG, Bacillus Calmette–Guérin; BW, birth weight; CMV, cytomegalovirus; COL, colistin; CPAP, continuous positive airway pressure; CRP, C-reactive protein; CSF, cerebrospinal fluid; EOL, early-onset listeriosis; EUCAST, European Committee on Antimicrobial Susceptibility Testing; GA, gestational age; GEN, gentamicin; HIE, hypoxic–ischemic encephalopathy; HSV, herpes simplex virus; IVH, intraventricular hemorrhage; MIC, minimum inhibitory concentration; NICU, neonatal intensive care unit; PCT, procalcitonin; PFGE, pulsed-field gel electrophoresis; PIP-TAZ, piperacillin–tazobactam; PLT, platelet count; PMA, postmenstrual age; SIMV, synchronized intermittent mandatory ventilation; SNHL, sensorineural hearing loss; TEOAE, transient evoked otoacoustic emissions; TMP-SMX, trimethoprim–sulfamethoxazole; WBC, white blood cell count; WGS, whole-genome sequencing.
