# Supplementary material for: Multifocal Early-Onset Neonatal Listeriosis with Discordant GradientStrip Ampicillin Non-Susceptibility: A Case Report
Source: Pathogens. 2026 Jun 26;15(7):674. doi: 10.3390/pathogens15070674 (PMC13414594; doi:10.3390/pathogens15070674)
Supplement: Supplementary file 1 [file pathogens-15-00674-s001.zip › pathogens-4379525-supplementary.pdf]

Supplementary Materials

All clinical events are reported with relative timing in days of life; Day 1 denotes the calendar day of birth. Calendar dates and exact times have been removed from the Supplementary Materials to maintain de-identification and consistency with the main manuscript, in accordance with CARE reporting principles and GDPR-related privacy considerations. Where multiple laboratory time-points existed on the same calendar day, values represent the same clinical laboratory set for that day. Values were verified against the individual laboratory reports and the institutional discharge letter.

Table S1. Chronological timeline of clinical events. ALT, alanine aminotransferase; AST, aspartate aminotransferase; BW, birth weight; Ca, calcium; CK, creatine kinase; CPAP, continuous positive airway pressure; CRP, C-reactive protein; CSF, cerebrospinal fluid; GA, gestational age; K, potassium; Mg, magnesium; NBIL, neonatal (total) bilirubin; NICU, neonatal intensive care unit; PCT, procalcitonin; PLT, platelet count; WBC, white blood cell count.

Day	Event
Day 1	Birth at outside facility (34 weeks GA, BW 1,990 g, Apgar 3 and 6 at 1 and 5 min). Resuscitation, CPAP, then intubation and mechanical ventilation for apneic episodes. Packed red blood cell microtransfusion (30 mL, O Rh-negative) administered for acute neonatal anemia in the context of severe early-onset sepsis with hemodynamic compromise.
Day 2 (admission at 30 h of life)	Transfer to tertiary NICU. Febrile (38.3 °C); ecchymoses; bilateral purulent ocular secretions. Empirical intravenous therapy initiated: piperacillin–tazobactam 80 mg/kg/dose (piperacillin component) every 6 h, gentamicin 5 mg/kg/dose every 36 h (extended-interval dosing appropriate for postmenstrual age 30–35 weeks), and colistimethate sodium 1.7 mg/kg/dose every 8 h (3.4 mg of colistin base activity per dose, ≈42,500 IU). Initial laboratory: PCT 111.3 ng/mL, CRP 159.6 mg/L, WBC 8.8 × 10^9/L, PLT 118 × 10^9/L (flagged for possible platelet aggregates), Hb 11.8 g/dL.
Day 4	Lumbar puncture: hemorrhagic CSF, culture sterile, negative bacterioscopy. Cranial ultrasound: diffuse cerebral hypoechogenicity (non-specific pattern; differential included hypoxic-ischemic encephalopathy, sepsis-associated cerebral edema, and early ischemic injury).
Day 5	Microbiological identification: *L. monocytogenes* from blood, gastric aspirate, pharyngeal exudate, ocular secretion, and skin swab. Gradient-strip MIC testing (ampicillin and meropenem on Liofilchem strips; erythromycin and trimethoprim–sulfamethoxazole on bioMérieux *Etest* strips; unconfirmed by broth microdilution): susceptible to meropenem and erythromycin; resistant to ampicillin and trimethoprim–sulfamethoxazole. Severe thrombocytopenia (PLT nadir 39 × 10^9/L). Antibiotic regimen modified: colistin discontinued, ampicillin–sulbactam (2:1) added (intravenous; ampicillin 50 mg/kg/dose every 12 h, increased to 75 mg/kg/dose every 12 h after postnatal age 7 days; planned 14 days); piperacillin–tazobactam and gentamicin continued during overlap (Days 5–7). CRP peak 230.0 mg/L; PCT 27.36 ng/mL (declining from 111.3 on Day 2); WBC 20.7 × 10^9/L

	(early leukocytosis). CK 251 U/L; transient unconjugated hyperbilirubinemia (NBIL 6.8 mg/dL); hypokalemia (K 3.0 mmol/L).
Day 7	Inflammatory markers improving: PCT 5.58 ng/mL, CRP 66.3 mg/L. Persistent leukocytosis (WBC 25.4 × 10^9/L). Platelets recovering (PLT 76 × 10^9/L from nadir 39 × 10^9/L on Day 5). Transaminases declining (AST 113 → 65 → 38 U/L; ALT 61 → 43 → 32 U/L across Days 2, 5, and 7). Persistent hypokalemia (K 3.0 mmol/L) with concurrent hypomagnesemia (Mg 1.2 mg/dL); electrolyte supplementation continued. Discontinuation of piperacillin–tazobactam and gentamicin. Enteral feeding initiated by orogastric gavage with preterm formula.
Day 9	Endotracheal aspirate culture sterile. Successful extubation; transition to non-invasive CPAP. Echocardiography: minor patent foramen ovale, Grade I tricuspid regurgitation, closed ductus arteriosus. Follow-up blood culture obtained (subsequently sterile at 48 h and 7 d; final validation Day 16).
Day 10	Inflammatory markers further improved: PCT 1.02 ng/mL, CRP 16.2 mg/L. WBC peak 49.7 × 10^9/L with marked left shift (18% bands, 5% metamyelocytes). Platelets normalized (273 × 10^9/L). Mild transaminase rebound (AST 48 U/L; ALT 30 U/L). Unconjugated hyperbilirubinemia resolved (NBIL 1.2 mg/dL).
Day 13	PCT 0.10 ng/mL, CRP 7.4 mg/L; inflammatory syndrome resolved. Vitamin D_3 prophylaxis initiated.
Day 14	Oxygen therapy discontinued.
Day 17	Cranial ultrasound: resolving right germinal-matrix/subependymal hemorrhage with cystic organization, compatible with prior Grade I hemorrhage.
Day 18	Completion of 14-day ampicillin–sulbactam course (Days 5–18). The single follow-up blood culture obtained on Day 9 had remained sterile through 48 h and 7 d incubation; final validation Day 16.
Day 20	Reticulocyte count 6/1,000 (0.6%; below lower limit of normal of 15/1,000), confirming hypoproliferative anemia. Reactive thrombocytosis (PLT 528 × 10^9/L), consistent with the typical post-septic rebound. Incidental mild hypercalcemia (Ca 11.1 mg/dL), seven days after initiation of vitamin D_3 prophylaxis; not repeated in the available record. Hemoglobin 9.4 g/dL.
Day 22	Oral iron supplementation initiated for residual anemia.
Day 27	Transfer back to referring hospital for continued convalescent care. Weight 2,440 g, length 48 cm, head circumference 32.5 cm. Hemoglobin 8.3 g/dL (residual anemia); platelet count 433 × 10^9/L. Stable cardiopulmonary status, full enteral feeding (50 mL × 8 meals/day), normal stooling and urinary output.

Table S2. Longitudinal hematological, biochemical, coagulation, and inflammatory marker profile.

Laboratory parameter	Day 2	Day 3	Day 5	Day 7	Day 10	Day 13	Day 20	Day 27	Unit	Reference interval
**Complete blood count**										
WBC	8.8	8.3	20.7	25.4	49.7	18.5	11.6	8.6	$\times 10^9$/L	6.0–17.5
RBC	3.49	3.09	4.20	3.80	3.80	3.12	3.11	2.70	$\times 10^{12}$/L	3.9–5.9
Hemoglobin*	11.8	10.7	13.2	11.8	11.8	10.2	9.4	8.3	g/dL	13.4–19.8
Hematocrit*	35.7	31.6	39.4	35.2	35.0	28.9	28.2	24.4	%	41.0–65.0
MCV	102	102	93.8	92.6	92.1	92.6	90.7	90.4	fL	88.0–140.0
MCH	33.8	34.6	31.4	31.1	31.1	32.7	30.2	30.7	pg	30.0–37.0
MCHC	33.1	33.9	33.5	33.5	33.7	35.3	33.3	34.0	g/dL	28.0–35.0
RDW	15.8	16.0	18.7	18.9	18.7	17.9	18.3	18.2	%	11.5–14.5
Platelet count	118†	89	39	76	273	294	528	433	$\times 10^9$/L	150–450
Plateletcrit	0.06	0.05	0.03	0.06	0.16	0.17	0.34	0.24	%	0.08–1.0
MPV	5.5	5.7	7.7	7.3	5.9	5.7	6.4	5.5	fL	6.0–10.0
PDW	19.7	19.8	19.4	19.5	19.1	19.1	18.1	18.3	%	10.0–18.0
**Leukocyte differential**										
Band neutrophils	2	4	—	—	18	—	—	—	%	1–4
Segmented neutrophils	92	78	—	—	56	46	—	—	%	30–70
Lymphocytes	4	12	—	—	20	48	—	—	%	20–40
Eosinophils	—	—	—	—	—	2	—	—	%	1–2
Monocytes	2	6	—	—	1	4	—	—	%	2–8
Metamyelocytes	—	—	—	—	5	—	—	—	%	0
**Hematology**										
Reticulocyte count	—	—	—	—	—	—	6	—	‰	15–35
**Coagulation**										

Laboratory parameter	Day 2	Day 3	Day 5	Day 7	Day 10	Day 13	Day 20	Day 27	Unit	Reference interval
Fibrinogen	421	—	316	256	262	—	—	—	mg/dL	130–330
**Clinical chemistry**										
Creatine kinase	—	—	251	—	—	—	—	—	U/L	35–145
ALT (GPT)	61	—	43	32	30	—	—	—	U/L	1–25
AST (GOT)	113	—	65	38	48	—	—	—	U/L	14–36
Direct bilirubin	—	—	0.0	—	0.0	—	—	—	mg/dL	0.0–0.3
Total bilirubin (unconjugated)‡	—	—	6.8	—	1.2	—	—	—	mg/dL	0.0–1.4
Calcium*	7.5	—	8.5	8.4	—	—	11.1	—	mg/dL	8.4–10.2
Creatinine*	0.9	—	0.7	0.5	0.4	0.3	0.3	—	mg/dL	0.70–1.20
Chloride	93	—	108	110	108	99	—	—	mmol/L	98–107
Glucose	57	—	87	73	94	63	—	—	mg/dL	65–110
Magnesium	1.9	—	1.7	1.2	—	—	—	—	mg/dL	1.6–2.3
Potassium	4.4	—	3.0	3.0	3.8	3.7	—	—	mmol/L	3.6–5.0
Sodium	125	—	133	137	139	132	—	—	mmol/L	137–145
Urea	50.1	—	44.3	62.1	50.9	50.8	51.3	—	mg/dL	15–36
C-reactive protein	159.6	—	230.0	66.3	16.2	7.4	—	—	mg/L	1–9
**Immunology**										
Procalcitonin§	111.3	—	27.36	5.58	1.02	0.10	—	—	ng/mL	<0.5 (institutional/IFCC); see footnote §

* Reference intervals are as reported by the institutional laboratory information system and are not all age-adjusted for the preterm neonate; values outside these intervals should be interpreted in the postnatal-age-specific clinical context. This applies particularly to hemoglobin and hematocrit (postnatal physiological nadir is substantially lower in preterm than in term infants), serum calcium (neonatal reference values differ from adult values), and creatinine (the early postnatal decline reflects clearance of maternal creatinine and is physiological).

† Initial platelet count of 118 × 10^9/L was flagged by the laboratory for possible microclots and platelet aggregates and should be interpreted cautiously; the subsequent Day 5 nadir of 39 × 10^9/L is free of pre-analytical artefact.

‡ Direct (conjugated) bilirubin was 0.0 mg/dL at both available time-points; the institutional "neonatal bilirubin" assay returned values identical to the indirect (unconjugated) fraction and is therefore reported as a single row.

§ Procalcitonin interpretation by IFCC categorical tiers: <0.5 ng/mL, low likelihood of bacterial infection; 0.5–<2 ng/mL, viral infection, chronic inflammation, or SIRS; 2–<10 ng/mL, possible systemic infection; ≥10 ng/mL, severe bacterial septicaemia or septic shock. Physiological postnatal PCT elevation may occur in the first 24–48 h of life; however, the Day 2 value of 111.3 ng/mL substantially exceeds the expected physiological range and supports bacterial sepsis.

Additional cerebrospinal fluid biochemical and cytological assessment.

Day	Assessment	Result	Unit / Comment
Day 4	CSF sample quality and cytology	Hemorrhagic CSF with clot present; cytology could not be processed	Lumbar puncture performed after ~48 h of empirical antimicrobial therapy
Day 4	CSF glucose	63	mg/dL
Day 4	CSF protein	4.46	g/L (= 446 mg/dL)
Day 4	CSF chloride	106	mmol/L
Day 4	CSF bacterioscopy and culture	Bacterioscopy negative; culture sterile	Sensitivity reduced by prior antimicrobial therapy

Abbreviations: ALT, alanine aminotransferase; AST, aspartate aminotransferase; CSF, cerebrospinal fluid; GOT, glutamic-oxaloacetic transaminase (synonym for AST); GPT, glutamic-pyruvic transaminase (synonym for ALT); IFCC, International Federation of Clinical Chemistry and Laboratory Medicine; MCH, mean corpuscular hemoglobin; MCHC, mean corpuscular hemoglobin concentration; MCV, mean corpuscular volume; MPV, mean platelet volume; PCT, procalcitonin (immunological marker; not to be confused with plateletcrit in the complete blood count); PDW, platelet distribution width; RBC, red blood cell count; RDW, red cell distribution width; SIRS, systemic inflammatory response syndrome; WBC, white blood cell count.

Table S3. Longitudinal microbiological assessment during hospitalization.

Day	Microbiological assessment	Biological specimen	Method	Result	Antimicrobial susceptibility
Day 2	Blood culture	Blood	Conventional blood-culture system; VITEK® 2 Compact automated identification system (bioMérieux, Marcy-l'Étoile, France) for species identification; gradient-strip MIC testing (Liofilchem for ampicillin and meropenem; bioMérieux Etest® for erythromycin and	Positive blood culture flagged on Day 4. Microscopy: erythrocytes, Gram-positive coccoid elements, and short Gram-positive rods arranged in angular forms (consistent with *Listeria* spp.). Identification: *Listeria monocytogenes*. The Gram-positive coccoid elements observed on the initial smear were not subsequently recovered or identified to species and are noted as an interpretive caveat in the main manuscript (§2.4.1).	Susceptible: meropenem, erythromycin. Resistant: ampicillin, trimethoprim–sulfamethoxazole. gradient-strip-reported and unconfirmed #.

Day	Microbiological assessment	Biological specimen	Method	Result	Antimicrobial susceptibility
			trimethoprim–sulfamethoxazole) for antimicrobial susceptibility		
Day 2	Gastric aspirate culture	Gastric aspirate	VITEK® 2 Compact automated identification system (bioMérieux, Marcy-l'Étoile, France); species-level identification only — antimicrobial susceptibility testing not performed	Positive for *Listeria monocytogenes*	—
Day 2	Pharyngeal exudate culture	Pharyngeal swab/exudate	VITEK® 2 Compact automated identification system (bioMérieux, Marcy-l'Étoile, France); species-level identification only — antimicrobial susceptibility testing not performed	Positive for *Listeria monocytogenes*	—
Day 2	Skin culture	Skin/tegument swab	VITEK® 2 Compact automated identification system (bioMérieux, Marcy-l'Étoile, France); species-level identification only — antimicrobial susceptibility testing not performed	Positive for *Listeria monocytogenes*	—
Day 2	Ocular secretion culture	Ocular secretion	VITEK® 2 Compact automated identification system (bioMérieux, Marcy-l'Étoile, France); gradient-strip MIC testing (Liofilchem for ampicillin and meropenem; bioMérieux Etest® for erythromycin and	Identification: *Listeria monocytogenes*. The same species was identified at all five sampled sites (blood, gastric aspirate, pharyngeal exudate, ocular secretion, skin swab). Molecular strain typing (PFGE or whole-genome sequencing) was not performed; therefore, clonal identity across sites cannot be confirmed.	Susceptible: meropenem, erythromycin. Resistant: ampicillin, trimethoprim–sulfamethoxazole. gradient-strip-reported and unconfirmed #.

Day	Microbiological assessment	Biological specimen	Method	Result	Antimicrobial susceptibility
			trimethoprim–sulfamethoxazole) for antimicrobial susceptibility		
Day 2	External auditory canal culture (bacterial)	External auditory canal swab	Standard bacterial culture on routine media; species-level identification only if pathogen growth obtained	Sparse polymorphic saprophytic Gram-positive flora; *Listeria monocytogenes* not recovered.	—
Day 4	Cerebrospinal fluid culture	Cerebrospinal fluid	Automated bacteriological method	Microscopy: frequent erythrocytes and relatively frequent polymorphonuclear leukocytes (consistent with hemorrhagic sample). Bacterioscopy negative; culture sterile. CSF sampling was performed after ~48 h of empirical antimicrobial therapy, which reduces culture sensitivity.	—
Day 9	Endotracheal aspirate culture	Endotracheal aspirate	Automated bacteriological method	Negative; 0 CFU/mL	—
Day 9	Follow-up blood culture	Blood	Conventional blood-culture system	Negative; sterile at 48 h preliminary reading and at 7-day final reading. Definitively validated on Day 16. Only a single follow-up blood culture was obtained, which is acknowledged as a limitation in the main manuscript (§3.5).	—

Susceptibility results are reported by gradient-strip MIC testing as recovered from the laboratory information system. The identification platform (VITEK® 2 Compact, bioMérieux) and the gradient-strip products (Liofilchem MIC Test Strip and Ezy MIC™ for ampicillin and meropenem, respectively; bioMérieux Etest® for erythromycin and trimethoprim–sulfamethoxazole) were used. Confirmatory broth microdilution against EUCAST breakpoints was not performed; the reported phenotype must therefore be interpreted as gradient-strip-reported and unconfirmed rather than as established acquired resistance.

Abbreviations: CFU, colony-forming units; CSF, cerebrospinal fluid; Etest, gradient-strip minimum inhibitory concentration test; EUCAST, European Committee on Antimicrobial Susceptibility Testing; h, hours; MIC, minimum inhibitory concentration; PFGE, pulsed-field gel electrophoresis.

Table S4. TORCH serological profile.

Day	Assessment	Biological specimen	Method	Result	Laboratory cut-off and interpretation
Day 5	*Toxoplasma gondii* IgM	Blood	Automated ELISA	0.11 S/CO	Negative <1; equivocal 1–1.2; positive >1.2 S/CO. Result: negative.
Day 5	Rubella virus IgM	Blood	Automated ELISA	0.11 S/CO	Negative <1; equivocal 1–1.2; positive >1.2 S/CO. Result: negative.
Day 5	Cytomegalovirus IgM	Blood	Automated ELISA	0.12 S/CO	Negative <1; equivocal 1–1.2; positive >1.2 S/CO. Result: negative.
Day 5	*Toxoplasma gondii* IgG	Blood	Automated ELISA	44 IU/mL	Negative <50 IU/mL; positive >50 IU/mL. Result: negative per institutional cut-off.
Day 5	Rubella virus IgG	Blood	Automated ELISA	24 IU/mL	Negative <10 IU/mL; positive >10 IU/mL. Result: positive per institutional cut-off; interpreted as transplacentally acquired maternal antibody.
Day 5	Cytomegalovirus IgG	Blood	Automated ELISA	15 IU/mL	Institutional cut-off reported as negative <0.5 IU/mL, positive >0.5 IU/mL. Result: positive per institutional cut-off; interpreted as transplacentally acquired maternal antibody.

Note. Herpes simplex virus serology and HSV PCR were not performed and empirical acyclovir was not initiated.

Abbreviations: ELISA, enzyme-linked immunosorbent assay; HSV, herpes simplex virus; Ig, immunoglobulin; IU, international units; PCR, polymerase chain reaction; S/CO, signal-to-cut-off ratio; TORCH, *Toxoplasma*, other (e.g., syphilis), *Rubella*, cytomegalovirus, herpes simplex virus.
